# Supplementary material for: Are carbon emissions trading and green financial instruments synergistic? -Comprehensive quantitative research based on content analysis
Source: PLoS One. 2024 Mar 7;19(3):e0298601. doi: 10.1371/journal.pone.0298601 (PMC10919601; doi:10.1371/journal.pone.0298601)
Supplement: S1 Data — (ZIP) [file pone.0298601.s001.zip › Data/地级市Z_F工作报告.zip!/╣≤╓▌/░▓╦│╩╨/└┤╫╘╣1⁄2╓┌║┼í╛┬φ┐╦╩2╛▌═°í┐_2017.docx]

各位代表：

现在，我代表市人民政府向大会作工作报告，请予审议，并请市政协委员和其他列席会议的同志提出意见。

一、2016年及本届政府工作回顾

2016年是“十三五”的开局之年，也是本届政府履职的收官之年。我们深入学习贯彻习近平总书记系列重要讲话精神特别是视察贵州时的重要讲话精神，在省委、省政府和市委的坚强领导下，按照“守底线、走新路、奔小康”的工作总纲，坚持主基调主战略，全力打好“一二四”攻坚战，以开局就是决战、起步就要冲刺的劲头，扎实苦干、锐意进取，经济社会保持快速健康发展，为决战决胜脱贫攻坚全面建成小康社会奠定了坚实基础。

预计，全市实现地区生产总值700.39亿元，增长12.4%；500万元以上固定资产投资652.15亿元，增长21.8%；工业增加值172.4亿元，增长11.2%；社会消费品零售总额176.34亿元，增长13.2%；财政总收入110.6亿元，增长13%，一般公共预算收入75.32亿元，增长13%；金融机构存款余额1090亿元，增长28%，贷款余额700亿元，增长15%；城镇和农村居民人均可支配收入25004元和8157元，分别增长9%、10.2%。在全省经济发展综合测评中排第六位。

一年来，我们主要做了以下工作：

一是突出大扶贫大数据两大战略行动，全面小康扎实推进。着力补齐脱贫攻坚短板，全年减少8.63万农村贫困人口，西秀区按国定标准退出，西秀区、平坝区通过同步小康达标验收，镇宁自治县及坡贡镇等3个贫困乡镇按省定标准“减贫摘帽”，162个贫困村出列，简嘎乡、大营镇两个极贫乡镇脱贫攻坚工作扎实推进。全力抓好易地扶贫搬迁，建成安置点52个、搬迁29784人。成功承办全国滇桂黔石漠化片区区域发展和扶贫攻坚现场推进会，争取到水利部与省政府在我市共建石漠化片区水利精准扶贫示范区。积极抢占大数据先机，全年完成信息基础设施投资10亿元，城域网上链带宽达到350Gbps，光网覆盖家庭158万户，光缆线路总长6.5万公里，3G/LTE基站总数8900个。中国联通智慧城市研发基地、旅游信息化孵化基地、大数据实验室基地落户安顺，智能手机、液晶显示器等项目落地建设，安顺大数据产业发展中心等产业平台初具规模，蚂蚁众创空间入选第三批国家级众创空间，软通动力、国电南自等企业不断发展壮大。西秀、平坝进入国家级电子商务示范县，普定、镇宁、关岭进入省级电子商务示范县。开展政府数据“聚通用”攻坚会战，组建安顺大数据公司，依托“云上贵州”系统平台，整合电子政务云、智慧旅游云、健康医疗云、扶贫云、广电云等数据资源，推进政府数据开放平台建设，提高大数据政用民用商用水平。以大数据为引领的电子信息产业产值达到125亿元，增长25%。

二是突出固定资产投资招商引资两个关键，发展后劲日益增强。全力推进项目建设，成功承办3次全省项目观摩会，先后组织5次项目集中开工，实施重大工程和重点项目470个，完成投资761亿元。沪昆客专安顺段建成通车，境内三个站场全面建成，安六城际铁路建设扎实推进。花安、安紫、普织高速公路加快建设，黄铺大道、关花大道、贵安大道开发区至黄果树段等骨干道路建设进展顺利。黄果树机场新开通西安、海口、西双版纳等3条航线，旅客吞吐量突破24万人次。黄家湾水利枢纽工程开工建设，新场河水库大坝封顶，老营、三岔河、纳井田等水库加快建设。强力推进招商引资，加大驻外招商工作力度，引进中国水电、内蒙伊利、武汉卓尔等33家500强企业来安投资。成功在青岛、深圳、重庆等地举办大型推介活动，签约项目202个，签约资金1224亿元。全年招商引资到位资金2018.79亿元，增长30.6%。

三是突出“新型城镇化+”工作主线，城乡建设亮点纷呈。编制实施《安顺市新型城镇化综合试点实施方案》，顺利通过国家新型城镇化综合试点年度评估。中心城区加快建设，历史文化街区改造全面启动，安顺高铁新城、虹山湖新区、平坝高铁新区初具规模，普定新城、镇宁环翠新区、关岭顶云新区、紫云新城建设扎实推进。杨武、乐平、丁旗、花江等一批小城镇蓬勃发展，完成城镇固定资产投资521.8亿元，增长37.3%。以承办全省改善农村人居环境工作大会为契机，成功打造大坝村、下九溪村等一批美丽乡村。旧州镇入选全国第一批特色小镇，秀水村被评为中国现代新村。

四是突出产业提质增效，发展质量不断提高。大力推进工业项目建设，完成500万元以上工业投资160亿元，实施“千企改造”项目110个，云雀新能源汽车、香雪海冷柜、馨思雅卫生用品等一批项目建成投产。推动产业园区转型升级，安顺高新区通过国家级高新区评估，西秀工业园区列入全国18个重点支持的循环化改造园区。新兴产业加快发展，高技术制造业增加值占规模以上工业增加值比重达16.5%，排名全省第一。军民融合取得新进展，与黎阳动力签订战略合作协议，全国“军工+”座谈会在安顺召开。规模以上工业实现增加值170亿元，增长12%。大力推进国家全域旅游示范区创建，开展旅游资源大普查，组建黄果树旅游区，“黄、龙、屯”实现一体化发展。围绕旅游主要客源地深入开展宣传营销，全市接待游客5655万人次，增长45%；实现旅游总收入560亿元，增长48%。以旅游为龙头的现代服务业加快发展，第三产业实现增加值349.07亿元，增长14.5%。大力发展现代山地特色高效农业，蔬菜、茶叶、水果、中药材、烤烟、优质粮油产业质量效益显著提升，实施“关岭牛振兴、蛋禽倍增、生猪提升”三大计划，畜牧业增加值占农林牧渔业增加值比重达到26.9%，位居全省第一。扶持壮大新型农业经营主体，发展各类农民专业合作社2594家、家庭农场97家、规模以上农产品加工企业201家。第一产业实现增加值124.07亿元，增长6.3%。

五是突出生态文明建设，生态环境有效改善。扎实推进“绿色安顺”建设三年行动计划，大力实施退耕还林等生态工程，完成营造林41万亩，治理水土流失面积220平方公里，综合治理石漠化面积507平方公里，建成10个城区山体公园（游园），森林覆盖率达到50%。强化节能减排工作，单位GDP能耗下降3.2%，超额完成省下达目标任务。推进粉煤灰、秸秆综合利用，固体废物综合利用率达95%以上。加强城镇污水和生活垃圾治理，市污水处理厂一期、二期工程升级改造达到一级A标，三期工程投入运行，建成一批生活垃圾收运设施，城镇污水处理率和生活垃圾无害化处理率分别达到85.3%、83.05%。加强水资源保护和大气污染治理，中心城区、县城饮用水源地水质达标率100%，中心城区环境空气质量达标率99.4%。

六是突出深化改革开放，发展活力更加强劲。推进供给侧结构性改革，消化房地产库存160万平方米，降低工业企业用电成本2亿元。“营改增”试点工作全面推开，减轻税负2.5亿元。深化政银企合作，地方金融机构“五个全覆盖”工程扎实推进，出台“微保贷”“中小企业创业贷”等扶持政策。积极推动黄果树旅游集团公司在主板上市，黄果树智慧旅游公司即将在新三板挂牌。不动产统一登记工作稳步推进。全面开展“三权”促“三变”改革，有效盘活农村资源资产资金，涌现出塘约、沙湾等一批改革亮点。全面扩大对外开放，“1+5”平台建设扎实推进。青岛对口帮扶工作深入开展，安顺-青岛产业园加快建设，园区产业中心项目主体完工。加强对外贸易合作，公用型保税仓建成投用，完成外贸进出口总额2.5亿美元，增长35.1%；实际利用外资1.58亿美元，增长30.6%。

七是突出增进民生福祉，社会事业加快发展。大力保障和改善民生，完成2016年“十件实事”。城镇新增就业5.25万人，新增创业3145户，新增创业带动就业10383人。大力提升城乡基本医疗、基本养老保障水平，城镇和农村低保标准分别提高10%和18.6%。实施棚户区改造60054户、农村危房改造20496户。大力发展教育事业，加快推进“新两基”工作，所有县区顺利通过“普十五”省级督导评估，市一中改扩建工程启动建设，安顺职院新校区建成搬迁，安顺学院通过教育部本科教学工作合格评估。着力提升卫生计生服务水平，市人民医院新院基本建成，市妇幼保健院（儿童医院）建成投用，三○二医院成功创建“三甲”医院，一批乡镇卫生院、村卫生计生室提标扩容，全面二孩政策顺利推进。加快发展文化体育事业，市博物馆基本建成，市奥体中心建成投用。加强和创新社会治理，60%的城市社区达到国家建设标准，完成“天网”三期工程，群众安全感和满意度分别达到96.05%、97.28%。安全生产和食品药品安全形势总体稳定。持续开展国防教育和拥军优属工作，国防后备力量建设取得新成绩。民族宗教、外事侨务、史志档案、人防、气象、老龄等各项工作取得新进展。

各位代表，2016年各项任务的完成，标志着市三届人大一次会议确定的五年奋斗目标全面实现。回顾本届政府履职这五年，我们团结和依靠全市各族人民，勠力同心、苦干实干，胜利完成“十二五”规划目标任务，创造了安顺发展历史上极不平凡的辉煌成就，在全面小康伟大征途上迈出了坚实步伐。

——五年锐意进取，安顺综合实力实现了大提升。我们坚持把提速度、增总量、升位次作为首要任务，始终以项目建设为总抓手，持续不断扩大投资规模，千方百计保持经济快速增长。全市地区生产总值占全省比重由5.4%上升到6%，年均增速达14.6%，高于全省平均水平，2014、2015两年增速排名全省第一。固定资产投资年均增速达46.2%，增速连续保持全省前列。一般公共预算收入年均增速达24.4%，2014、2015两年增速排名全省第一。在全省经济发展综合测评中，2013至2015年连续三年位居全省第五，摆脱了长期挂末的被动局面。

——五年奋力拼搏，安顺产业升级实现了大发展。我们坚持把转方式、调结构、促转型作为根本之策，每年由县区争办全市工业项目推进大会、旅游产业发展大会、现代山地农业观摩会，形成发展倒逼机制，推动“赶”“转”并举，促进三次产业新旧动能加快转换。工业质量效益双提升，规模以上工业增加值实现翻番，年均增速达14%。改造提升煤、电等传统产业，大力发展纺织、造纸、制药等特色轻工业，轻重工业比从14.4∶85.6调整为23.8∶76.2。军民融合深入推进，新能源汽车、民用航空等装备制造业加快发展。大数据、大健康、新型建筑建材等新兴产业增加值占生产总值比重逐年提高。成功举办第一届、第二届贵州（安顺）国际石材博览会，石博会永久落户安顺。旅游业发展呈现井喷态势，接待游客量年均增长28.4%、旅游收入年均增长29.8%。打造大屯堡等一批旅游景区景点，全市4A级旅游景区从1个增加到7个。旅游设施提档升级，建成了百灵希尔顿逸林、万绿城铂瑞兹等一批高星级酒店。旅游业态不断丰富，农旅、文旅深度融合，浪塘、小河湾、秀水、高荡、三合苗寨、桃子等乡村旅游发展迅速，推出“大明屯堡”大型实景剧。格凸河攀岩、坝陵河低空跳伞、镇宁半程马拉松等国际赛事影响力逐步提升，成功承办了第十届全省旅游产业发展大会。金融保险、商贸物流、邮电通信、信息服务、酒店餐饮等现代服务业快速发展，黄桶-幺铺物流园区加快建设。农业产业结构持续优化，全市82个涉农乡镇实现农业园区全覆盖，建成省级农业园区28个，农业“接二连三”步伐不断加快，粮经比从52∶48调整为30∶70，畜牧业增加值占农林牧渔业增加值比重连续五年位居全省前列。

——五年不懈努力，安顺城乡面貌实现了大变化。我们坚持把“新型城镇化+”作为工作主线，积极推广“一分三向”安顺模式，着力完善中心城区、县城和重点镇、美丽乡村三级载体基础设施和公共服务设施，深入推进户籍制度改革，全市城镇化率提高9个百分点，达到47.5%。平坝完成撤县设区，普定撤县设区工作进展顺利，“一城三区”城市框架初步形成，主城区面积拓展到90平方公里。每年举办新型城镇化推进暨小城镇建设发展大会，城乡建设快速推进，一批城市综合体拔地而起，一批特色小城镇活力四射，一批美丽乡村欣欣向荣。公路、铁路、航空等基础设施不断完善，贵安大道、安普大道、二环路等多条城市快速干道建成通车，沪昆客专全线贯通，安顺进入高铁时代，黄果树机场通航城市达到10个，水道、慢道、轨道“三道”工程加快建设，黄果树悬挂式轨道交通项目启动实施。创建工作取得阶段性成果，成功创建全国双拥模范城、省级森林城市，全国文明城市创建通过年度测评，国家卫生城市创建通过暗访考核，国家园林城市创建通过省级预检，国家环保模范城市创建通过省级预评估。

——五年开拓创新，安顺改革开放实现了大跨越。我们坚持把改革开放作为关键一招，不断深化改革挖潜力、扩大开放增活力。重点领域和关键环节改革实现突破，供给侧结构性改革取得实效。持续深化国企改革，完善国企负责人薪酬制度、外派监事会制度。混合所有制改革取得新进展，组建油脂集团、轴承联盟等一批混合所有制企业。大力扶持民营经济发展，全市民营经济市场主体达12.25万户，注册资本金922.03亿元，民营经济增加值占生产总值比重达65%，连续五年位居全省第一。平台公司发展壮大，通过信托、融资租赁、基金、公司债、企业债等方式拓宽融资渠道，直接融资比例大幅提升。大力发展村级集体经济，“空壳村”全面消除。教育、科技、文化、卫生等领域改革深入推进。多领域、全方位、深层次扩大开放，一批开放平台和载体建设取得突破。组织参加贵州·北京大数据产业发展推介会、茶博会、文博会、绿博会、酒博会、水博会、民博会、贵洽会等活动。招商引资成效明显，大数据、服装、汽车、军民融合等领域招商取得新突破，一批500强企业来安投资兴业。

——五年克难攻坚，安顺社会民生实现了大改善。我们坚持把改善和保障民生作为工作的出发点和落脚点，一大批事关群众切身利益的热点难点问题得到有效解决。深入实施“32768”扶贫攻坚行动计划，认真落实“1+10”配套政策措施，扎实推进脱贫攻坚“五个一批”“十一项行动”重大项目，累计减少37.32万农村贫困人口。始终把就学、就医、就业作为最大的民生保障，建成一批学校、医院等公共服务设施，城镇登记失业率控制在4.2%以内，城乡居民收入持续增加，社会保障水平显著提高。大力发展文化体育事业，推进文艺精品创作，不断丰富群众文化生活，成功举办贵州省第三届农民运动会、安顺市第一届运动会。加强和创新社会治理，“平安安顺”建设深入推进，人民群众安全感和满意度大幅提升，荣获“平安市”称号。

过去五年，我们坚持依法行政，不断完善依法行政制度体系,着力转变政府职能，积极推进“放管服”改革，实施权力清单和责任清单制度，取消市级行政审批事项69项。各县区全部建成政务服务中心，行政审批事项全部进驻中心办理，率先接入贵州省网上办事大厅统一行政审批系统。我们自觉接受市人大的法律监督、工作监督和市政协的民主监督，办理人大代表建议337件、政协提案935件，办复率100%，认真听取各民主党派、工商联和无党派人士的意见建议，积极支持工会、共青团、妇联、文联、科协、残联、侨联、红十字会、计生协会等依法按章履行职责。深入推进干部作风建设，着力整治“四风”问题，认真抓好督促整改，廉洁建设进一步加强。

各位代表，五年的成就令人振奋。这是省委、省政府和市委坚强领导的结果，是市人大依法监督和市政协民主监督的结果，是全市各族人民奋发进取、共同努力的结果。在此，我谨代表市人民政府，向全市各族人民，向各位代表、政协委员，向各民主党派、工商联、无党派人士、人民团体，向驻安人民解放军、武警消防官兵、公安政法干警、中央和省驻安单位，向老同志及所有关心、支持我市改革发展和现代化建设的各界人士，表示衷心的感谢并致以崇高的敬意！

在看到成绩的同时，我们也清醒地认识到，全市发展仍面临不少困难，与人民群众的期待还有不小差距。主要表现在：经济综合实力还不强，群众生活水平还不高，保障和改善民生的任务依然繁重；产业结构不合理，产业优势不突出，转方式调结构的任务仍然艰巨；交通、信息、城乡基础设施支撑能力不足，瓶颈制约因素还需全力破解；城乡发展还存在差距，住房保障、教育、医疗、养老等公共服务还存在“短板”；农村地区贫困人口多、贫困程度深，脱贫攻坚难度大；政府自身建设还存在薄弱环节，一些工作人员法治意识、责任意识、服务意识不够，作风建设和廉洁建设需要进一步加强。我们将牢记职责使命，加倍努力工作，切实解决好这些问题。

二、今后五年的目标任务

市第四次党代会明确提出，今后五年工作的总体要求是：高举中国特色社会主义伟大旗帜，坚持以马克思列宁主义、毛泽东思想、邓小平理论、“三个代表”重要思想、科学发展观为指导，深入学习贯彻习近平总书记系列重要讲话精神，认真贯彻落实党中央治国理政新理念新思想新战略，紧密团结在以习近平同志为核心的党中央周围，统筹推进“五位一体”总体布局，协调推进“四个全面”战略布局，按照“守底线、走新路、奔小康”的工作总纲，坚持主基调主战略，实施大扶贫、大数据两大战略行动，以“新型城镇化+”为主线，突出全域旅游、军民融合，抓好脱贫攻坚、产业发展、生态建设，持续打好“一二四”攻坚战，发挥比较优势，推动黔中崛起，倾力打造山地特色新型城镇化示范区，凝心聚力抓发展，建设秀美新安顺，为决战决胜脱贫攻坚全面建成小康社会而努力奋斗！

今后五年经济社会发展的主要预期目标是:全市生产总值年均增长14%左右，固定资产投资年均增长25%以上，规模以上工业增加值年均增长12%以上，社会消费品零售总额年均增长13%以上，一般公共预算收入年均增长15%以上，城乡居民人均可支配收入年均增长分别达到13%左右和15%以上，城镇化率达到60%以上，森林覆盖率达到60%以上。

今后五年，是我市实现弯道取直、跨越发展的关键时期，也是可以大有作为、必须奋发有为的重要战略机遇期，我们要坚定战略部署不动摇、咬定目标任务不放松，大力实施“六大工程”，着力构建“六大体系”。

（一）实施“美丽家园工程”，构建具有山地特色的新型城镇化和城乡统筹发展体系

坚持以人的城镇化为核心，把握好“特”“新”“化”的新型城镇化丰富内涵，按照“全域安顺、产城互动、景城一体、城乡统筹”的发展方向，完成新型城镇化综合试点任务，全面推行“一分三向”安顺模式，形成层次分明、特色鲜明的城、镇、村发展体系，倾力打造山地特色新型城镇化示范区。做大做强中心城市，实现普定撤县设区，形成“一城三区”的中心城市框架，城市空间拓展到220平方公里以上，建成百万人口生态旅游城市。规划建设平坝-普定-镇宁、主城区至紫云城市快速干道，加快观安路、北斗湖路等与贵安新区互联互通道路建设，建成贵安大道开发区至黄果树段。推进镇宁融入中心城区发展，推动关岭、紫云建设成为区域性中心城市。抓好50个重点示范小城镇建设，建成一批具有产业支撑、富有地域特色、人居环境良好的小城镇。持续推进“四在农家·美丽乡村”建设，大力改善农村人居环境，建成100个以上特色美丽乡村，实现一村一景、一村一特、村景相连，各美其美、美美与共。加强城市形象设计，延续历史文脉，彰显地域特色，打响城市品牌。推进海绵城市和地下综合管廊建设。加快水道、慢道、轨道“三道”建设，把城镇、乡村、景区连成一体，构建亲近宜人的滨水景观系统、快进慢游的休闲慢行系统、多元体验的轨道交通系统。

（二）实施“产业提升工程”，构建符合资源禀赋和市场需求的现代特色产业体系

大力发展新型工业，构筑产业之柱。弘扬工匠精神，深入实施《中国制造2025》，推进军民融合发展，建设军民融合创新示范区，积极发展民用航空等高端装备制造业。改造提升煤、电、铝等传统产业，培育壮大茶、酒、饮用水、蜡染、服装纺织、特色食品、民族医药等特色轻工业，大力发展大数据、大健康、新型建筑建材等新兴产业，打造能源原材料、军民融合、智能终端3个百亿级产业。全面推进产业园区错位发展、提质升级，建设3个千亿级园区、2个百亿级园区。大力扶持企业发展，形成大型企业带动力强、中型企业成长性好、小微企业特色鲜明的协同发展格局。实现规模以上工业增加值年均增长12%以上。大力发展以旅游为龙头的现代服务业，激活产业之脉。加强旅游基础设施建设，推进旅游与农业、工业、文化、体育、大扶贫、大数据、大健康等深度融合，丰富旅游业态，建成国家全域旅游示范区，实现山水观光游向休闲体验游升级、景区景点游向全域旅游升级、过境游向目的地游升级，打造国际知名、国内一流山地旅游目的地。推进服务业聚集区建设，推动生产性服务业向专业化和价值链高端延伸、生活性服务业向精细化和高品质转变。实现服务业增加值年均增长15%。大力发展现代山地特色高效农业，夯实产业之基。持续推动农业结构调整，做大做强蔬菜、茶叶、水果、中药材等特色种植，加快发展现代山地生态畜牧业，畜牧业增加值占农林牧渔业增加值比重达到34%以上。深化“乡乡镇镇建农业园区”工程，建成一批以整县为单位的国家级和省级现代农业示范区。大力扶持培育新型农业经营主体，实现村村有农民专业合作经济组织。提高农产品精深加工水平，发展农村电子商务，加快农产品市场体系建设，促进农业“接二连三”。实现第一产业增加值年均增长8%以上。

（三）实施“强基固本工程”，构建适应经济社会发展的现代基础设施体系

树立布局合理、适度超前、延伸乡村的理念，完善现代基础设施，提升综合承载能力。加强交通基础设施建设，构建“三横一纵五射线”铁路网络体系，建成普织、紫望等高速公路，全市高速公路通车里程达到580公里，加大国省道改造力度，农村公路总里程达1.1万公里以上，构建乡村公路互联互通网络，实现组组通油路（水泥路）。完成黄果树机场改扩建工程，加密国内航班，开通国际航线。加强内河航运及码头升级改造，打通黔中出海通道。加强水利基础设施建设，建成黄家湾水利枢纽等一批大中小型骨干水源工程，实现县县有中型水库、乡乡有稳定供水水源，农村自来水普及率达到80%以上、水质达标率达到90%以上。加强电力规划建设，提高城乡供电保障能力和供电质量。加强信息基础设施建设，推进5G网络建设应用，实施骨干网络优化工程、城镇网络覆盖工程、农村宽带延伸工程，建设“无线城市”，基本实现城镇公共区域免费WiFi全覆盖，实现所有自然村通宽带。

（四）实施“绿色生态工程”，构建可持续发展的生态环境安全保障体系

秀美安顺，拒绝污染，要牢牢守住发展和生态两条底线，将绿色理念贯穿生产生活生态全领域全过程，深化生态文明体制改革，大力发展绿色经济、建设绿色家园、健全绿色制度、构筑绿色屏障、培育绿色文化，全力建设国家生态文明示范区。实施青山、蓝天、碧水、净土工程，大力开展荒山造林、封山育林、退耕还林、石漠化综合治理，推进高速铁路、高速公路、城际干道沿线生态景观长廊建设，加强森林、湿地保护，抓好水土保持和水生态修复。加强资源节约综合利用，大力发展新能源，强化节能减排指标约束，实施大气、水、土壤污染防治行动计划，中心城区环境空气质量达标率实现巩固提升，重点流域水质优良率达到92%以上，县城、城市污水处理率分别达到85%、95%以上，垃圾无害化处理率达到90%以上。

（五）实施“活力聚增工程”，构建推动跨越发展的动能支撑体系

坚持战略导向、问题导向、民生导向，全面深化改革。充分发挥市场在资源配置中的决定性作用，深化商事制度、财税金融、投融资等领域改革，加快完善现代市场体系，不断激发市场活力和创造力。扎实推进供给侧结构性改革，提升要素资源配置效率。深化国有企业改革，健全完善现代企业制度。积极发展混合所有制经济。支持民营经济加快发展，破除民间资本进入基础设施、社会事业等领域体制机制障碍，全市民营经济增加值占生产总值比重提高到70%。深化农村综合改革，加快城乡要素平等交换和公共资源均衡配置。深化公共事业领域改革，加快教育、医疗、文化和社会治理等领域改革步伐，促进社会公平正义。实施创新驱动发展战略，深入推进大众创业万众创新，加大科技人才培养和引进力度，促进科技创新与产业链的有机衔接。推进“1+5”开放平台建设，构筑扩大开放的新高地。坚持“走出去”和“引进来”并重，主动融入“一带一路”、长江经济带、珠江-西江经济带发展，进一步加大招商引资、招才引智力度。深化与青岛等友好城市的交流合作，推进安顺-贵安-贵阳经济一体化发展。

（六）实施“民生幸福工程”，构建惠及广大人民群众的基本公共服务体系

深入实施大扶贫战略行动，坚持精准扶贫、精准脱贫基本方略，全面消除绝对贫困。聚焦群众关切点，推进基本公共服务均等化，提高覆盖面和供给质量，提升人民群众的获得感和幸福感。着力扩大创业就业，织密社会保障网底，提高社会保险统筹层次和保障水平。着力改善居住环境，切实解决城乡困难群体的住房问题。大力实施教育优先发展战略，加强师资队伍建设，提升教育质量，不断缩小城乡教育差距，逐步构建适应经济社会发展需要的现代教育体系。完善城乡医疗卫生和公共卫生服务体系，积极发展中医药、民族医药，全面推动卫生计生事业大发展、大提升、大跨越，打造健康安顺。建立健全公共文化体育服务体系，推动民族特色文化繁荣发展，培育壮大文化产业。深入推进社会主义精神文明建设，倡导全民阅读，加强科学普及，提高全民文明素养和科学素质。创新基层社会服务，完善公共安全体系和安全生产体系，强化社会治安综合治理，建设更加和谐稳定的社会环境。

各位代表，蓝图已绘就，奋进正当时。实现伟大的理想，没有平坦的大道可走，在新的征途上，需要我们一步一个脚印继续奋勇前进。我们一定不忘初心、不负重托，团结全市各族人民为之共同奋斗。我们坚信，290万勤劳智慧的安顺人民一定会创造出无愧于历史的新业绩，谱写出幸福美满生活的新篇章!

三、2017年重点工作

2017年是新一届政府履职的开局之年，是实施“十三五”规划的关键之年，是全面建成小康社会的突破之年，我们要按照中央、全省经济工作会议精神，坚持稳中求进工作总基调，牢固树立和贯彻落实新发展理念，适应把握引领经济发展新常态，全面做好稳增长、促改革、调结构、惠民生、防风险各项工作。

2017年全市经济社会发展的主要预期目标是：地区生产总值增长13%以上，500万元以上固定资产投资增长22%以上，工业增加值增长12%以上，一般公共预算收入增长10.5%以上，社会消费品零售总额增长13%，城镇和农村居民人均可支配收入分别增长11%和13%，城镇登记失业率控制在4.2%以内，居民消费价格涨幅3%左右，城镇化率达到51%，完成省下达的节能减排降碳目标任务。

围绕实现上述目标，重点抓好以下八个方面工作：

（一）全力打造山地特色新型城镇化示范区，促进“五型城市”加快建设

全面推进试点工作。推广“一分三向”安顺模式，深入推进“多规合一”，建立“多规融合”信息化管理平台。深化户籍制度改革，落实“1+12”文件体系，引导人口有序转移，着力解决好转移人口就业问题。加快建立农业转移人口市民化成本分担机制、多元化可持续的城镇化投融资机制。加强新型城镇化项目库建设，实施新型城镇化融资规划，启动建设一批示范性项目。

全面加强城乡建设。实施《安顺市城市总体规划（2016-2030年）》，力争完成普定撤县设区，完善“一城三区”中心城市框架。大力推进历史文化街区改造，加快安顺高铁新城、虹山湖新区、平坝高铁新区、普定新城等城市新区建设，推进镇宁环翠、关岭顶云、紫云新城等一批重点新区建设。推广“旧州经验”，大力实施小城镇建设“8+X”项目，抓好30个特色示范小城镇建设。深入推进“四在农家·美丽乡村”六项小康行动计划，大力实施改善农村人居环境“10+N”工程，结合乡村历史传承和山水风貌，建成20个以上特色各异的美丽乡村。加快“三道”工程建设，完成贯城河滨水景观改造一期工程，建成人行慢道32公里、骑行道26公里，黄果树悬挂式轨道交通项目开通运行，启动实施高铁安顺西站经龙宫至黄果树旅游扶贫小火车项目。加快海绵城市和地下综合管廊建设，建成主城区雨水园示范工程12处、雨水河示范工程30公里、地下综合管廊试点工程1处。支持平坝办好第七届安顺市新型城镇化推进暨小城镇建设发展大会。

全面提升管理水平。完成城市管理综合执法体制改革。实施道路畅通工程，打通各类“断头路”，推进城市交通智能管理平台建设，在主城区新建一批智能化基础设施，有效缓解交通拥堵状况。优先发展公共交通，新开通公交线路4条以上，加强出租车和网约车运营管理。实施城市绿化美化亮化工程，加大公路沿线、城区背街小巷综合治理力度，有效整治横穿马路、噪音污染、占道经营等现象，努力提升城市“颜值”。

（二）全力建设军民融合创新示范区，促进工业发展转型升级

强力推进军民融合。抢抓军民融合上升为国家战略的重大机遇，推动军转民、民参军，促进军地技术、人才的有效转换和资源共享。编制实施军民融合产业发展规划，加快构建“一核一带”空间布局，争创以航空产业为主体的国家级军民融合创新示范区。力争安顺国家高新区获国务院批准，加快安顺民用航空产业国家高技术产业基地发展。大力推进平坝黎阳航空小镇、乐平通用机场建设。深化与贵飞、安大、风雷、安吉等军工企业合作，重点发展高级教练机、无人机、通用飞机、航空零部件、燃气轮机、专用车辆、医用氧舱，加快虹轴精密轴承、云雀新能源汽车、云马新能源汽车、湘黔非晶合金变压器等军民融合项目建设。

强力推进大数据战略行动。实施信息基础设施三年会战，完成信息基础设施投资10亿元。加快4G网络建设，实施“光网安顺”和“宽带乡村”工程，实现全市自然村光网全覆盖。加快推动部门政务平台互联互通，开展政府数据“聚通用”攻坚会战，政府公共数据资源实现100%上云。加快中国联通智慧城市、旅游信息化、大数据实验室三大基地建设，大力发展VR产品、智能手机、3D打印机、液晶面板等智能终端产品，实现以大数据为引领的电子信息产业产值150亿元。

强力推进工业发展。实施100个重点工业项目，完成工业投资190亿元。加快推进“百企引进”“千企改造”工程和“双培育”“双退出”行动计划，大力实施“百千万”工程，抓好百灵制药、安酒集团、晨春石材、兴贵恒远等一批重点企业改造工程，完成技改投资46亿元。改造提升煤炭、电力、化工、特色轻工等传统产业，启动黄果树铝业“煤电铝一体化”项目，支持垃圾发电等节能环保项目发展。培育壮大石材产业，建成西南国际生态石材博览交易中心，办好第三届贵州（安顺）国际石材博览会。加快大健康新医药产业发展，实施一批高水平大健康产业项目，支持兴东等大健康企业加快发展。做大做强西秀产业园，加快普定、镇宁、关岭、紫云工业园区建设，实现园区工业总产值650亿元。全年实现规模以上工业增加值192亿元，增长12%以上。

（三）全力创建全域旅游示范区，促进现代服务业繁荣发展

加快全域旅游发展。深入推进黄果树、龙宫、屯堡、格凸河景区管理一体化进程。加快推进大屯堡、格凸河景区创建国家5A级旅游景区，继续做好黄果树·屯堡景观申报世界自然与文化双遗产工作。挖掘屯堡文化、民族文化、红色文化、“三线”文化等内涵，打造提升一批休闲度假、康体养生、山地观光、户外运动、低空飞行、汽车露营、科普探险、民族文化演艺及避暑、温泉、滑雪等新兴业态。启动坝陵河生态户外运动基地建设，积极争取国家航空飞行营地落户安顺。大力开发特色食品、民族民间工艺品等旅游商品。设立旅游发展基金，力争黄果树旅游集团公司在主板上市，培育壮大本土旅游市场主体，积极引进国内外优强旅游企业。大力开展旅游宣传推介，办好黄果树瀑布节、格凸河国际攀岩、坝陵河国际跳伞、镇宁国际半程马拉松等节庆赛事活动，支持关岭办好第四届安顺旅游产业发展大会。全年接待游客7630万人次，增长35%；实现旅游总收入770亿元，增长38%。

加快完善旅游配套设施。推进宁龙黄旅游公路建设，启动实施紫黄旅游公路、马路场至宁谷旅游慢道工程，规划建设覆盖旧州、天龙、云峰等屯堡景区的旅游环线公路。依托高铁站点和重要交通节点，加快旅游集散中心等服务平台建设。加大航线培育力度，争取新开通3条航线，旅客吞吐量突破30万人次。积极推进高端酒店、精品酒店、主题酒店、特色民宿等建设，提升旅游接待服务能力。

加快发展现代服务业。全面推进黄桶-幺铺物流园区建设，基本建成园区主干路网，园区公路港、物流港建设初具规模。支持黔中、川渝、豪德等物流园区发展，引导专业批发市场合理布局、有序发展，完善中心城区商业区规划，高水平打造商业综合体。加快推进重点现代服务业集聚区建设，力争创建2个省级现代服务业集聚区。推动现代金融、商务服务、研发设计、会展广告、信息服务等生产性服务业创新发展，推进商贸、家政、住宿餐饮、社区照料、医患陪护等生活性服务业加快发展，实现服务业增加值增长15%以上。

（四）全力发展现代山地特色高效农业，促进农民增收致富

积极调整农业产业结构。深入推进农业供给侧结构性改革。实施最严格的耕地保护制度，稳定粮食生产，落实粮食安全责任制，确保粮食安全。以安顺国家农业科技园区、现代高效农业示范园区、高速公路产业带及农业高新技术示范点为平台，加快特色优势产业发展，粮食、蔬菜、水果、茶叶、中药材种植面积分别达到215万亩、180万亩、90万亩、80万亩、50万亩，收购烤烟20万担。加快山地生态畜牧业发展，畜牧业增加值占农林牧渔业增加值比重达到28%以上。深化农工、农旅融合，大力发展金刺梨、火龙果、葡萄和生猪、肉牛、禽蛋等农畜产品精深加工及乡村旅游。扩大“三品一标”生产规模，培育一批具有市场竞争力的特色农产品品牌。

积极推进农村各项改革。继续深化农村“三权”促“三变”改革，健全完善“1+N”配套政策，盘活农村资源资产资金，壮大农村集体经济，激发农村发展活力。大力推广“塘约道路”“秀水模式”“大坝经验”。积极推进“七权”同确试点，完成全市所有乡镇农村土地承包经营权确权登记颁证和国有林场改革工作，全面推进水务一体化改革。

积极拓宽农民增收渠道。大力发展农业产业化龙头企业、农民专业合作社、家庭农场等新型农业经营主体。积极发展农村电子商务，搭建农产品电子商务平台，促进“安货出山”。加快国家级、省级电子商务示范县建设，力争紫云进入省级电子商务示范县。加大农民培训力度，实施新型职业农民培育、“雨露计划”等培训项目，培训农民2.5万人次，鼓励农民创业和务工。积极发展生态农业、休闲农业、观光农业、设施农业，拓宽农民增收渠道。

（五）全力加快基础设施建设，促进发展条件有效改善

大力构建现代综合交通体系。推进安六城际铁路建设，积极配合做好贵阳至兴义铁路、贵阳-安顺城际轨道交通项目前期工作，启动主城区城市轨道交通项目。建成花安、安紫高速公路，推进普织、紫望高速公路建设，开工建设都香高速（安顺段），争取普（定）盘（江）、镇（宁）安（龙）、贵阳大外环（平坝段）高速公路尽早开工。加快推进黄铺大道、关花大道、贵安大道开发区至黄果树段、平坝东外环线、北斗湖路、安宁大道等骨干道路建设。做好黄果树机场改扩建工程前期工作。完成光照、董箐等库区航运工程。

大力完善水利电力基础设施。推进黄家湾水利枢纽工程建设，建成老营、新场河、纳井田、三岔河等水库，扎实推进大洞口、坝陵河、木桥、打冲沟、芦坝、雨棚、白沙、堰坝口、小鲁灰、干沟等水库建设。新增有效灌溉面积2.4万亩，完成水利固定资产投资20亿元以上。加快新一轮农村电网改造升级，完成电力基础设施投资7亿元。

（六）全力加强生态文明建设，促进环境质量持续提升

加强生态建设。推进封山育林、植树造林和退耕还林，加强森林资源管护，治理水土流失面积220平方公里，综合治理石漠化面积100平方公里，森林覆盖率达到52%以上。加快邢江河、虹山湖、黄果树等湿地公园和安顺植物园建设。推进城镇公共绿地、环城林带、交通沿线及景区景点景观绿化建设，建成综合公园、山体公园、社区公园、街头游园（广场）35个，新增公园绿地200万平方米以上。推进国家森林城市创建工作，成功创建国家园林城市。

加强环境保护。推进生活垃圾、餐厨垃圾、建筑垃圾、医疗废弃物等综合处置，新建改造中心城区、县城、重点镇垃圾处理和收运设施16个，垃圾无害化处理率达到86.5%。加快建设主城区供水三期工程，新增污水处理规模1.5万吨/日，新建和改造污水管网50公里，县城以上污水处理厂达到国家一级A标排放标准，建制镇污水处理厂全部投入运营。稳步提升环境空气质量，抓好秸秆禁烧综合利用。加强农业面源污染治理。严格环境监管执法，依法严处各类环境违法行为。成功创建国家环保模范城市。

加强节能减排。加快淘汰落后产能，加强脱硫脱硝等环保设施运行管理，完成黄标车、老旧车和高污染燃料锅炉年度淘汰任务。加快清洁能源推广使用步伐，大力推进城镇燃气基础设施建设，开展乡镇管道燃气供应试点，建成电动汽车充电站4座、充电桩1117个。培育普及绿色理念，全面推行勤俭节约、绿色低碳、文明健康的生活方式。

（七）全力深化改革开放，促进发展活力不断增强

不断深化重点领域改革。扎实推进供给侧结构性改革，落实 “三去一降一补”各项政策措施。实施新一轮“民营经济倍增计划”，民营经济增加值占生产总值比重达到66%以上。深入推进不动产统一登记制度和市场主体“五证合一”、个体工商户“两证整合”登记制度改革。继续深化财税体制改革，提高财税管理科学化、精细化水平。扎实推进“营改增”工作，有效减轻企业税负压力。加大金融支持力度，大力实施“微保贷”“中小企业创业贷”等扶持政策，加快推进地方金融机构“五个全覆盖”工程，提升市级平台公司经营管理水平，进一步拓宽投融资渠道。深化科技体制改革，积极构建技术创新体系，鼓励支持新产品、新技术、新工艺的研发创新，推进科技成果有效转化，综合科技进步水平指数达到60%。

不断提高对外开放水平。继续推进以国家新型城镇化综合试点为引领的“1+5”平台建设，推动安顺经济技术开发区升级国家级开发区工作。深化与贵阳市、贵安新区的战略合作，积极抓好内陆开放型经济试验区建设。认真谋划“沪昆高铁经济带”，构建区域合作发展新格局。加强与青岛等发达城市的交流合作，加快安顺-青岛产业园建设，推动一批项目落户园区。积极加强与港澳台、东盟、加拿大、瑞士、韩国等的交流合作，让安顺的“朋友圈”越来越大。深入实施外贸进出口翻番计划，确保进出口总额增长15%，实际利用外资增长20%。继续加大招商引资力度，聚焦新型城镇化、全域旅游、军民融合、大数据、大健康等领域，精准引进更多高技术企业、龙头企业和优强企业，大力引进拟上市企业落户安顺，每个县区至少引进2家拟上市企业，全年确保完成省外3000万以上招商引资项目到位资金937.5亿元，增长25%以上。

不断发展壮大县域经济。全面提升县域经济发展内生动力和综合竞争力，突出区域特色，发挥比较优势，全力打造“创新西秀”，推动“平坝崛起”，做强“活力普定”，做大“开放镇宁”，做美“生态关岭”，建设“绿色紫云”，升级“军民融合开发区”，舞好“旅游龙头黄果树”。

（八）全力保障改善民生，促进社会和谐进步

着力推进大扶贫战略行动。贯彻落实《贵州省大扶贫条例》，扎实推进“32768”扶贫攻坚行动计划，认真落实“1+10”配套政策措施，打好易地扶贫搬迁关键一仗，突出抓好产业扶贫。全力推进大营镇、简嘎乡两个极贫乡镇脱贫攻坚工作。用足用好省脱贫攻坚投资基金，充分发挥资本市场助推脱贫攻坚的作用。推进省部共建石漠化片区水利精准扶贫示范区建设，扎实做好东西部扶贫协作和对口支援，深化青岛市、中航集团等对我市的帮扶工作。实现平坝区、普定县和137个贫困村按国定标准退出，紫云自治县和5个贫困乡镇按省定标准“减贫摘帽”。

着力推进社会事业发展。优先发展教育事业，深入实施“新两基”攻坚，平坝区、普定县县域义务教育均衡发展通过国家认定，推进市一中改扩建工程、安顺学院改扩建二期工程建设，全面建成安顺职院新校区并实现整体搬迁。深化城市公立医院综合改革和计划生育服务管理改革，建立完善分级诊疗制度，推进“百院大战”等六大医疗卫生机构标准化建设工程，市人民医院新院全面建成投用，市妇幼保健院成功创建“三甲”医院，推动市中医院创建“三甲”医院。积极建设市图书馆、文化馆、科技馆、档案馆、大剧院、工人文化宫、老年活动中心、青少年活动中心、妇女儿童活动中心等公共文化设施。推进就业创业促进工程，实施“雁归兴安”“锦绣计划”“千名大学生创业行动计划”。深入实施全民参保计划，有序推进机关事业单位养老保险改革，加快整合城乡居民基本医疗保险制度。统筹推进城乡社会救助体系建设。加强国防后备力量建设，支持驻安部队调整改革，巩固全国双拥模范城创建成果。加快发展慈善、老龄事业，进一步做好国防教育、民族宗教、新闻出版、广播影视、防灾减灾、史志、文物、档案、外事、侨务、对台等工作。

着力推进社会治理创新。加强城乡基层服务网络建设，完善社区配套设施和服务功能，推行社区网格化管理，创建和谐社区。推进安全生产领域改革发展，强化安全生产责任制，建立健全安全预防控制体系，推进安全生产形势持续稳定好转。加强食品药品安全监管。建立健全市、县、乡、村四级信访维稳工作信息网络，积极预防和妥善处置群体性、突发性事件。实施“天网”四期工程，建设覆盖全市范围的天网视频探头、卡口，构建全方位、立体化的监控系统，完善社会治安防控体系和矛盾纠纷多元化解体系，深化“平安细胞工程”，进一步提高人民群众的安全感和满意度。扎实开展创建工作，成功创建全国文明城市、国家卫生城市。

着力办好“十件实事”。①减少农村扶贫对象8万人以上；完成易地扶贫搬迁1.5万人。②城镇新增就业5.4万人，新增创业2800人，创业带动就业1.1万人。③城镇和农村居民最低生活保障标准在2016年基础上提高10%。④完成县乡道提等改造217公里；建成农村通组路640公里；实施农村10万人饮水安全巩固提升工程。⑤完成棚户区改造4.5万户，完成农村危房改造1万户。⑥新建和改造提升旅游公厕200个以上，全市所有旅游公厕达到Ａ级标准；新建改扩建标准化菜市场30个。⑦全市3G/LTE基站总数达到9000个，城域网上链带宽达到500Gbps以上，全市景区、车站、广场、机场、体育场、医院等公共区域实现免费WiFi全覆盖。⑧建成农村义务教育学校标准化食堂100个；实施农村学前教育儿童营养改善计划6.58万人。⑨提档升级乡镇卫生院（社区卫生服务中心）18所，建设乡镇中医馆40个、乡镇远程医疗会诊系统46个，改扩建村卫生计生服务室200个。⑩完成虹山湖景观整治三期工程建设;新增主城区公共停车位1000个。

四、全面提高政府治理服务能力

各位代表，面对新形势、适应新常态、实现新发展、建设新安顺，对政府工作提出了新的更高要求，我们要继往开来、砥砺前行，坚持从严治党，坚持依法行政，坚持创新管理，坚持主动作为，全面提高政府治理服务能力，努力建设人民满意的政府，决不辜负人大代表、政协委员的信任，决不辜负全市人民的重托。

（一）坚持从严治党，着力打造责任政府

一是增强“四个意识”。坚决维护以习近平同志为核心的党中央权威，切实增强政治意识、大局意识、核心意识、看齐意识，更加自觉地在思想上政治上行动上与以习近平同志为核心的党中央保持高度一致。认真贯彻《关于新形势下党内政治生活的若干准则》，全面落实政府党组主体责任，执行政府党组工作规则，落实双重组织生活制度。二是加强权力监督。严格执行《中国共产党党内监督条例》，加强行政监察和审计监督，持续深化拓展民生监督，不断完善政府权力运行监督机制。强化“一岗双责”，真管真严、敢管敢严、长管长严，依法规范行使权力，把权力关进制度的笼子。三是强化源头防腐。切实增强各级干部廉洁自律意识，认真贯彻中央八项规定、省委十项规定和市委实施意见，坚持不懈纠正“四风”，严厉整治各种违纪行为，以零容忍态度惩治腐败，大力营造风清气正的政务环境。

（二）坚持依法行政，着力打造法治政府

一是强化法治意识。坚持法定职责必须为、法无授权不可为，着力推进机构、职能、权限、程序、责任法定化。不断深化行政执法体制改革，稳妥实施事业单位改革。坚持用法治思维、法治方式开展工作，严格执行重大决策合法性审查制度。二是规范行政决策。坚持民主集中制原则，建立健全“三重一大”集体决策制度。加强与民主党派、工商联、无党派人士、人民团体的沟通协商，认真听取意见建议，完善人大代表议案建议和政协提案的办理联系机制。推动公众参与重大决策，健全公示听证、决策咨询、专家论证等制度，进一步提高政府决策的科学化、民主化、法治化水平。三是完善监督机制。依法接受市人大及其常委会的法律和工作监督，主动接受市政协和各民主党派的民主监督，重视司法、舆论、社会监督，提高政府工作透明度，让权力在阳光下运行。

（三）坚持创新管理，着力打造创新政府

一是创新行政管理方式。不断深化行政审批制度改革，大力推进简政放权，坚持运用市场化、社会化、信息化、透明化方式强化行政管理。全面推进政务公开，推进公共资源配置、重大项目批准和实施、公益事业建设等信息公开。二是创新公共服务供给。扩大政府购买公共服务，推广政府与社会资本合作模式，鼓励社会资本通过特许经营等方式参与公益性事业投资运营，提高公共服务供给效率。三是创新信息平台建设。推进“互联网+政务服务”，实现政务、商务、事务“三务合一”，公共资源交易办理规范化、交易服务信息化、交易风险归零化“三化一体”，电子政务网、政府门户网、网上办事大厅“三网互通”，构建“审批服务一站式、交易流程一标准、公共服务一条龙”的大服务格局。

（四）坚持主动作为，着力打造服务政府

一是牢记服务宗旨。始终坚持“一切要为人民打算，一切要为小康苦干”，树立勤政为民的理念，倾听群众呼声、关心群众冷暖、关注群众福祉，多为群众干实事、干好事。二是强化作风建设。持续推进作风建设常态化、长效化，加大问责力度，大力整治庸政懒政怠政现象，不断提高行政效能，努力营造想干事、能干事、干成事的良好氛围。坚持主动作为，加大跑上跑外力度，大兴调查研究之风，积极破解改革发展难题。三是狠抓工作落实。坚持目标引领，着力推进工作，真抓实干、务求实效，不断提高工作执行力和落实力，把心思用在干事业上，把精力投到抓落实中，以实干求业绩，以实绩赢民心。

各位代表，宏伟蓝图令人鼓舞，美好愿景催人奋进。新一届政府即将开启新的征程，面对新的目标任务，我们深感责任重大、使命光荣。让我们更加紧密地团结在以习近平同志为核心的党中央周围，在省委、省政府和市委的坚强领导下，振奋精神、勇于担当，凝心聚力抓发展，建设秀美新安顺，为决战决胜脱贫攻坚全面建成小康社会而努力奋斗！
